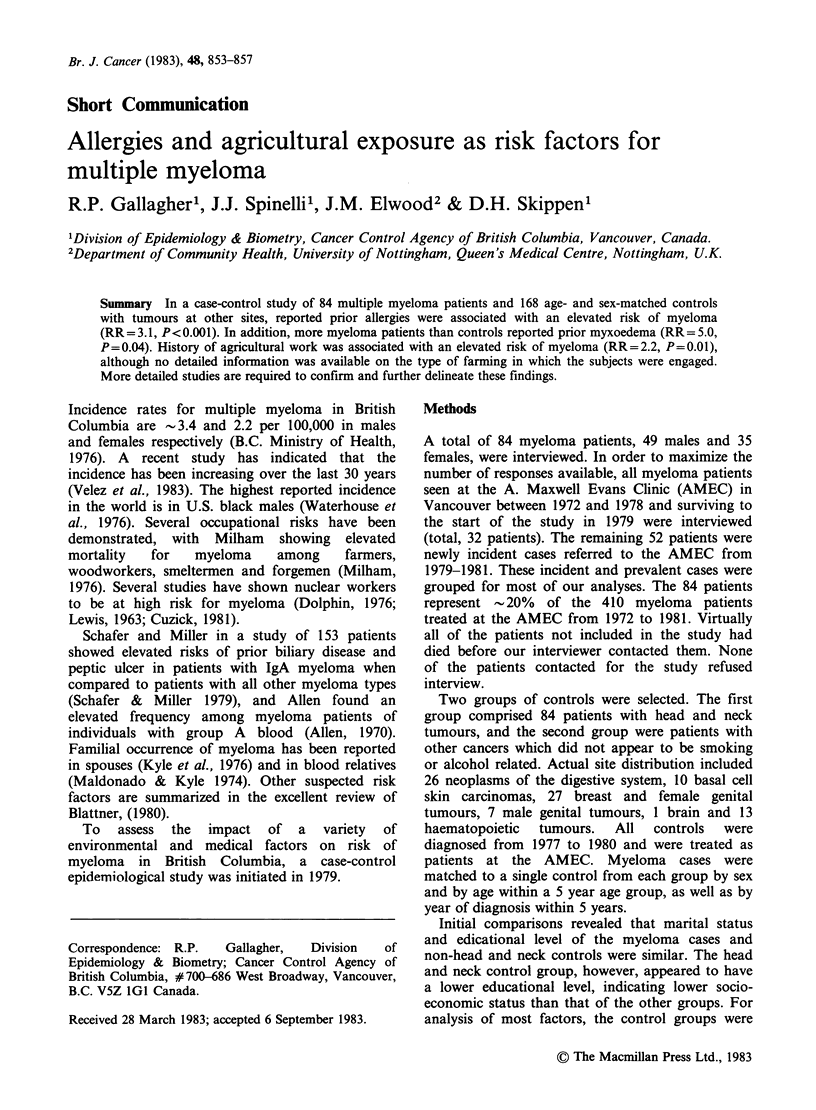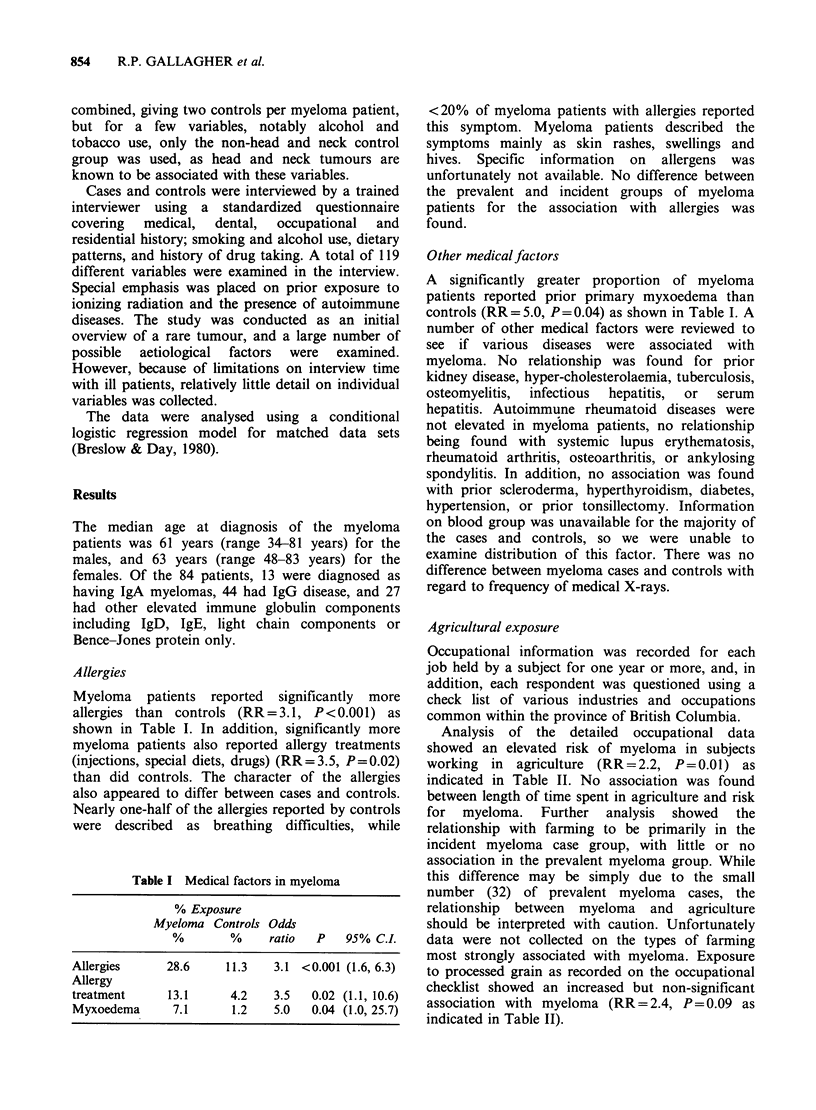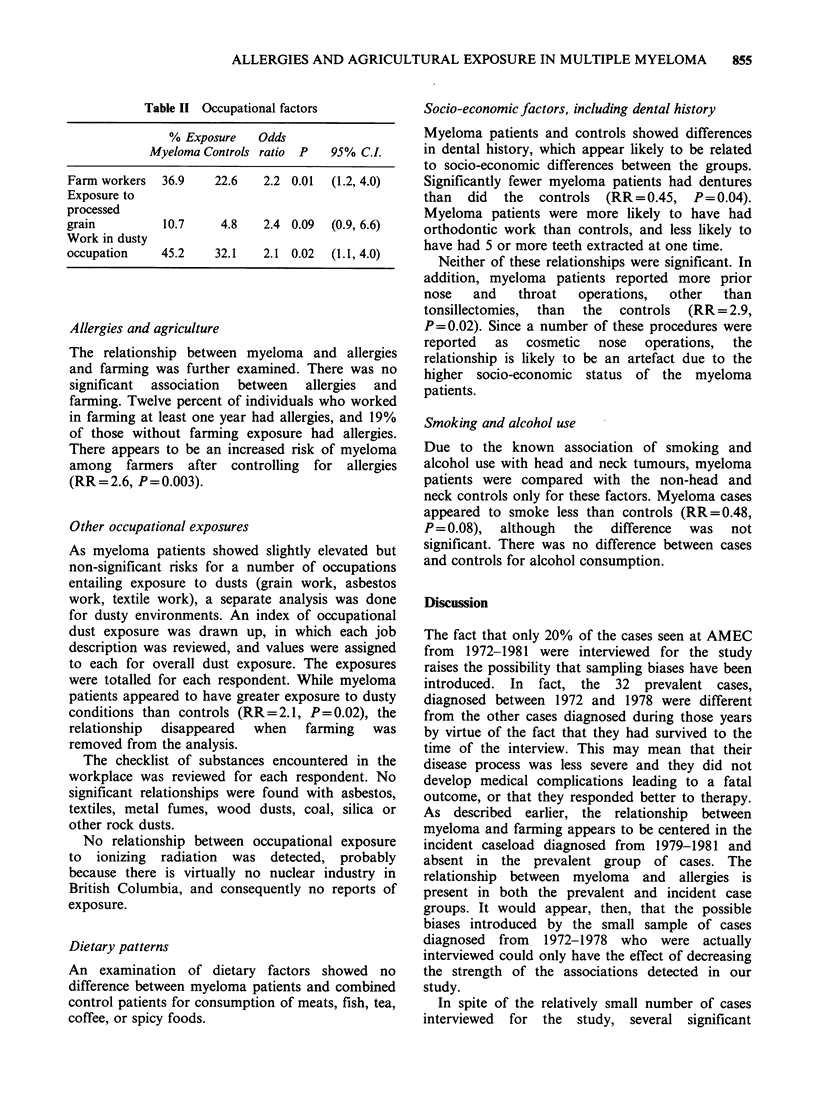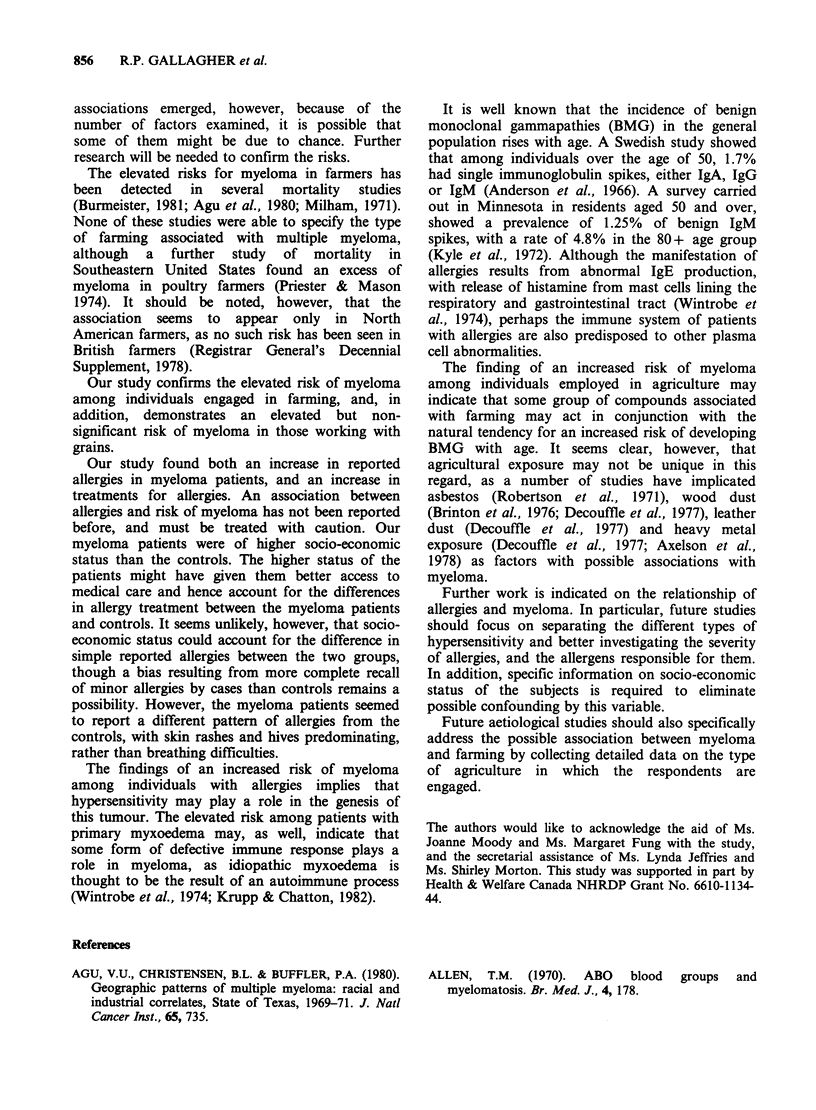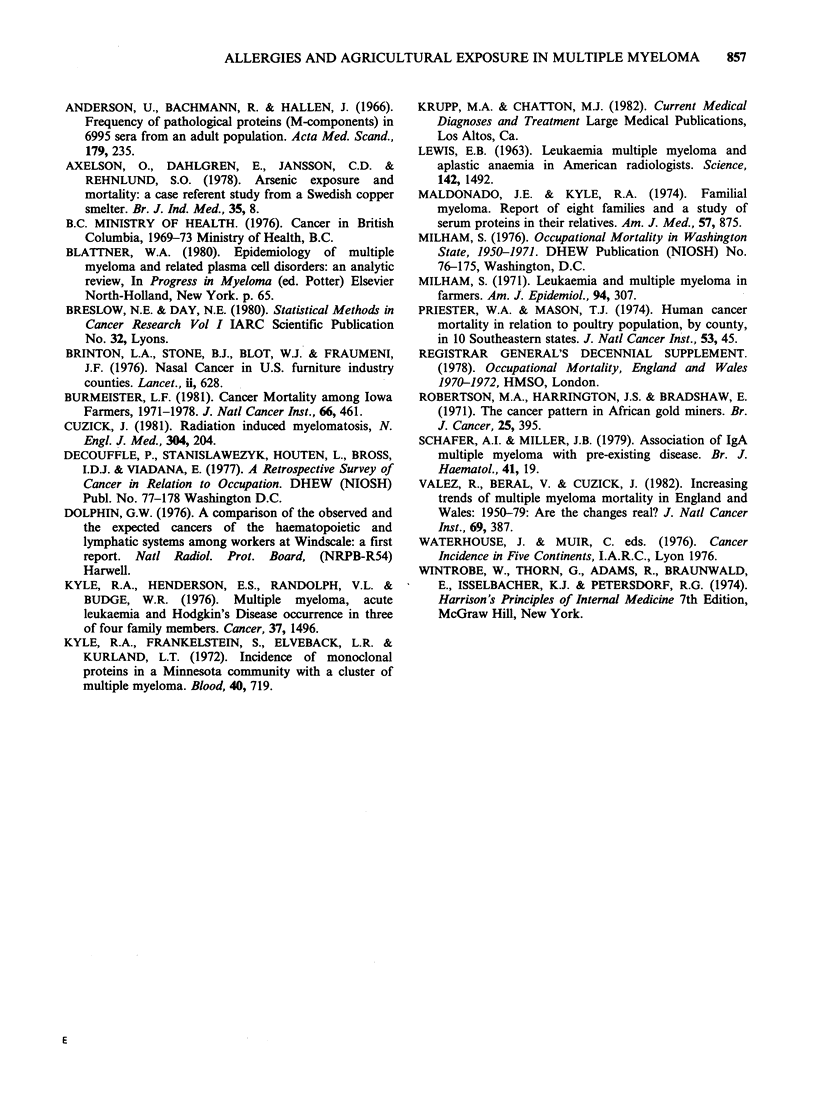# Allergies and agricultural exposure as risk factors for multiple myeloma.

**DOI:** 10.1038/bjc.1983.277

**Published:** 1983-12

**Authors:** R. P. Gallagher, J. J. Spinelli, J. M. Elwood, D. H. Skippen

## Abstract

In a case-control study of 84 multiple myeloma patients and 168 age- and sex-matched controls with tumours at other sites, reported prior allergies were associated with an elevated risk of myeloma (RR = 3.1, P less than 0.001). In addition, more myeloma patients than controls reported prior myxoedema (RR = 5.0, P = 0.04). History of agricultural work was associated with an elevated risk of myeloma (RR = 2.2, P = 0.01), although no detailed information was available on the type of farming in which the subjects were engaged. More detailed studies are required to confirm and further delineate these findings.


*Br. J. Cancer* (1983), **48**, 853–857

### Short Communication

### Allergies and agricultural exposure as risk factors for multiple myeloma

R.P. Gallagher[1], J.J. Spinelli[1], J.M. Elwood[2] & D.H. Skippen[1]

[1]*Division of Epidemiology & Biometry, Cancer Control Agency of British Columbia, Vancouver, Canada.*
[2]*Department of Community Health, University of Nottingham, Queen's Medical Centre, Nottingham, U.K.*

**Summary** In a case-control study of 84 multiple myeloma patients and 168 age- and sex-matched controls with tumours at other sites, reported prior allergies were associated with an elevated risk of myeloma (RR = 3.1, $P < 0.001$). In addition, more myeloma patients than controls reported prior myxoedema (RR = 5.0, $P = 0.04$). History of agricultural work was associated with an elevated risk of myeloma (RR = 2.2, $P = 0.01$), although no detailed information was available on the type of farming in which the subjects were engaged. More detailed studies are required to confirm and further delineate these findings.

Incidence rates for multiple myeloma in British Columbia are ~3.4 and 2.2 per 100,000 in males and females respectively (B.C. Ministry of Health, 1976). A recent study has indicated that the incidence has been increasing over the last 30 years (Velez *et al.*, 1983). The highest reported incidence in the world is in U.S. black males (Waterhouse *et al.*, 1976). Several occupational risks have been demonstrated, with Milham showing elevated mortality for myeloma among farmers, woodworkers, smeltermen and forgemen (Milham, 1976). Several studies have shown nuclear workers to be at high risk for myeloma (Dolphin, 1976; Lewis, 1963; Cuzick, 1981).

Schafer and Miller in a study of 153 patients showed elevated risks of prior biliary disease and peptic ulcer in patients with IgA myeloma when compared to patients with all other myeloma types (Schafer & Miller 1979), and Allen found an elevated frequency among myeloma patients of individuals with group A blood (Allen, 1970). Familial occurrence of myeloma has been reported in spouses (Kyle *et al.*, 1976) and in blood relatives (Maldonado & Kyle 1974). Other suspected risk factors are summarized in the excellent review of Blattner, (1980).

To assess the impact of a variety of environmental and medical factors on risk of myeloma in British Columbia, a case-control epidemiological study was initiated in 1979.

Correspondence: R.P. Gallagher, Division of Epidemiology & Biometry; Cancer Control Agency of British Columbia, #700–686 West Broadway, Vancouver, B.C. V5Z 1G1 Canada.

Received 28 March 1983; accepted 6 September 1983.

### Methods

A total of 84 myeloma patients, 49 males and 35 females, were interviewed. In order to maximize the number of responses available, all myeloma patients seen at the A. Maxwell Evans Clinic (AMEC) in Vancouver between 1972 and 1978 and surviving to the start of the study in 1979 were interviewed (total, 32 patients). The remaining 52 patients were newly incident cases referred to the AMEC from 1979–1981. These incident and prevalent cases were grouped for most of our analyses. The 84 patients represent ~20% of the 410 myeloma patients treated at the AMEC from 1972 to 1981. Virtually all of the patients not included in the study had died before our interviewer contacted them. None of the patients contacted for the study refused interview.

Two groups of controls were selected. The first group comprised 84 patients with head and neck tumours, and the second group were patients with other cancers which did not appear to be smoking or alcohol related. Actual site distribution included 26 neoplasms of the digestive system, 10 basal cell skin carcinomas, 27 breast and female genital tumours, 7 male genital tumours, 1 brain and 13 haematopoietic tumours. All controls were diagnosed from 1977 to 1980 and were treated as patients at the AMEC. Myeloma cases were matched to a single control from each group by sex and by age within a 5 year age group, as well as by year of diagnosis within 5 years.

Initial comparisons revealed that marital status and edicational level of the myeloma cases and non-head and neck controls were similar. The head and neck control group, however, appeared to have a lower educational level, indicating lower socio-economic status than that of the other groups. For analysis of most factors, the control groups were

© The Macmillan Press Ltd., 1983

combined, giving two controls per myeloma patient, but for a few variables, notably alcohol and tobacco use, only the non-head and neck control group was used, as head and neck tumours are known to be associated with these variables.

Cases and controls were interviewed by a trained interviewer using a standardized questionnaire covering medical, dental, occupational and residential history; smoking and alcohol use, dietary patterns, and history of drug taking. A total of 119 different variables were examined in the interview. Special emphasis was placed on prior exposure to ionizing radiation and the presence of autoimmune diseases. The study was conducted as an initial overview of a rare tumour, and a large number of possible aetiological factors were examined. However, because of limitations on interview time with ill patients, relatively little detail on individual variables was collected.

The data were analysed using a conditional logistic regression model for matched data sets (Breslow & Day, 1980).

### Results

The median age at diagnosis of the myeloma patients was 61 years (range 34–81 years) for the males, and 63 years (range 48–83 years) for the females. Of the 84 patients, 13 were diagnosed as having IgA myelomas, 44 had IgG disease, and 27 had other elevated immune globulin components including IgD, IgE, light chain components or Bence–Jones protein only.

### *Allergies*

Myeloma patients reported significantly more allergies than controls (RR = 3.1, $P<0.001$) as shown in Table I. In addition, significantly more myeloma patients also reported allergy treatments (injections, special diets, drugs) (RR = 3.5, $P=0.02$) than did controls. The character of the allergies also appeared to differ between cases and controls. Nearly one-half of the allergies reported by controls were described as breathing difficulties, while <20% of myeloma patients with allergies reported this symptom. Myeloma patients described the symptoms mainly as skin rashes, swellings and hives. Specific information on allergens was unfortunately not available. No difference between the prevalent and incident groups of myeloma patients for the association with allergies was found.

**Table I** Medical factors in myeloma

| | *% Exposure* Myeloma % | Controls % | *Odds ratio* | *P* | *95% C.I.* |
|---|---|---|---|---|---|
| Allergies | 28.6 | 11.3 | 3.1 | <0.001 | (1.6, 6.3) |
| Allergy treatment | 13.1 | 4.2 | 3.5 | 0.02 | (1.1, 10.6) |
| Myxoedema | 7.1 | 1.2 | 5.0 | 0.04 | (1.0, 25.7) |

### *Other medical factors*

A significantly greater proportion of myeloma patients reported prior primary myxoedema than controls (RR = 5.0, $P=0.04$) as shown in Table I. A number of other medical factors were reviewed to see if various diseases were associated with myeloma. No relationship was found for prior kidney disease, hyper-cholesterolaemia, tuberculosis, osteomyelitis, infectious hepatitis, or serum hepatitis. Autoimmune rheumatoid diseases were not elevated in myeloma patients, no relationship being found with systemic lupus erythematosis, rheumatoid arthritis, osteoarthritis, or ankylosing spondylitis. In addition, no association was found with prior scleroderma, hyperthyroidism, diabetes, hypertension, or prior tonsillectomy. Information on blood group was unavailable for the majority of the cases and controls, so we were unable to examine distribution of this factor. There was no difference between myeloma cases and controls with regard to frequency of medical X-rays.

### *Agricultural exposure*

Occupational information was recorded for each job held by a subject for one year or more, and, in addition, each respondent was questioned using a check list of various industries and occupations common within the province of British Columbia.

Analysis of the detailed occupational data showed an elevated risk of myeloma in subjects working in agriculture (RR = 2.2, $P=0.01$) as indicated in Table II. No association was found between length of time spent in agriculture and risk for myeloma. Further analysis showed the relationship with farming to be primarily in the incident myeloma case group, with little or no association in the prevalent myeloma group. While this difference may be simply due to the small number (32) of prevalent myeloma cases, the relationship between myeloma and agriculture should be interpreted with caution. Unfortunately data were not collected on the types of farming most strongly associated with myeloma. Exposure to processed grain as recorded on the occupational checklist showed an increased but non-significant association with myeloma (RR = 2.4, $P=0.09$ as indicated in Table II).

**Table II** Occupational factors

| | % Exposure Myeloma | Controls | Odds ratio | P | 95% C.I. |
|---|---|---|---|---|---|
| Farm workers | 36.9 | 22.6 | 2.2 | 0.01 | (1.2, 4.0) |
| Exposure to processed grain | 10.7 | 4.8 | 2.4 | 0.09 | (0.9, 6.6) |
| Work in dusty occupation | 45.2 | 32.1 | 2.1 | 0.02 | (1.1, 4.0) |

*Allergies and agriculture*

The relationship between myeloma and allergies and farming was further examined. There was no significant association between allergies and farming. Twelve percent of individuals who worked in farming at least one year had allergies, and 19% of those without farming exposure had allergies. There appears to be an increased risk of myeloma among farmers after controlling for allergies (RR=2.6, $P$=0.003).

*Other occupational exposures*

As myeloma patients showed slightly elevated but non-significant risks for a number of occupations entailing exposure to dusts (grain work, asbestos work, textile work), a separate analysis was done for dusty environments. An index of occupational dust exposure was drawn up, in which each job description was reviewed, and values were assigned to each for overall dust exposure. The exposures were totalled for each respondent. While myeloma patients appeared to have greater exposure to dusty conditions than controls (RR=2.1, $P$=0.02), the relationship disappeared when farming was removed from the analysis.

The checklist of substances encountered in the workplace was reviewed for each respondent. No significant relationships were found with asbestos, textiles, metal fumes, wood dusts, coal, silica or other rock dusts.

No relationship between occupational exposure to ionizing radiation was detected, probably because there is virtually no nuclear industry in British Columbia, and consequently no reports of exposure.

*Dietary patterns*

An examination of dietary factors showed no difference between myeloma patients and combined control patients for consumption of meats, fish, tea, coffee, or spicy foods.

*Socio-economic factors, including dental history*

Myeloma patients and controls showed differences in dental history, which appear likely to be related to socio-economic differences between the groups. Significantly fewer myeloma patients had dentures than did the controls (RR=0.45, $P$=0.04). Myeloma patients were more likely to have had orthodontic work than controls, and less likely to have had 5 or more teeth extracted at one time.

Neither of these relationships were significant. In addition, myeloma patients reported more prior nose and throat operations, other than tonsillectomies, than the controls (RR=2.9, $P$=0.02). Since a number of these procedures were reported as cosmetic nose operations, the relationship is likely to be an artefact due to the higher socio-economic status of the myeloma patients.

*Smoking and alcohol use*

Due to the known association of smoking and alcohol use with head and neck tumours, myeloma patients were compared with the non-head and neck controls only for these factors. Myeloma cases appeared to smoke less than controls (RR=0.48, $P$=0.08), although the difference was not significant. There was no difference between cases and controls for alcohol consumption.

### Discussion

The fact that only 20% of the cases seen at AMEC from 1972–1981 were interviewed for the study raises the possibility that sampling biases have been introduced. In fact, the 32 prevalent cases, diagnosed between 1972 and 1978 were different from the other cases diagnosed during those years by virtue of the fact that they had survived to the time of the interview. This may mean that their disease process was less severe and they did not develop medical complications leading to a fatal outcome, or that they responded better to therapy. As described earlier, the relationship between myeloma and farming appears to be centered in the incident caseload diagnosed from 1979–1981 and absent in the prevalent group of cases. The relationship between myeloma and allergies is present in both the prevalent and incident case groups. It would appear, then, that the possible biases introduced by the small sample of cases diagnosed from 1972–1978 who were actually interviewed could only have the effect of decreasing the strength of the associations detected in our study.

In spite of the relatively small number of cases interviewed for the study, several significant associations emerged, however, because of the number of factors examined, it is possible that some of them might be due to chance. Further research will be needed to confirm the risks.

The elevated risks for myeloma in farmers has been detected in several mortality studies (Burmeister, 1981; Agu *et al.*, 1980; Milham, 1971). None of these studies were able to specify the type of farming associated with multiple myeloma, although a further study of mortality in Southeastern United States found an excess of myeloma in poultry farmers (Priester & Mason 1974). It should be noted, however, that the association seems to appear only in North American farmers, as no such risk has been seen in British farmers (Registrar General's Decennial Supplement, 1978).

Our study confirms the elevated risk of myeloma among individuals engaged in farming, and, in addition, demonstrates an elevated but non-significant risk of myeloma in those working with grains.

Our study found both an increase in reported allergies in myeloma patients, and an increase in treatments for allergies. An association between allergies and risk of myeloma has not been reported before, and must be treated with caution. Our myeloma patients were of higher socio-economic status than the controls. The higher status of the patients might have given them better access to medical care and hence account for the differences in allergy treatment between the myeloma patients and controls. It seems unlikely, however, that socio-economic status could account for the difference in simple reported allergies between the two groups, though a bias resulting from more complete recall of minor allergies by cases than controls remains a possibility. However, the myeloma patients seemed to report a different pattern of allergies from the controls, with skin rashes and hives predominating, rather than breathing difficulties.

The findings of an increased risk of myeloma among individuals with allergies implies that hypersensitivity may play a role in the genesis of this tumour. The elevated risk among patients with primary myxoedema may, as well, indicate that some form of defective immune response plays a role in myeloma, as idiopathic myxoedema is thought to be the result of an autoimmune process (Wintrobe *et al.*, 1974; Krupp & Chatton, 1982).

It is well known that the incidence of benign monoclonal gammapathies (BMG) in the general population rises with age. A Swedish study showed that among individuals over the age of 50, 1.7% had single immunoglobulin spikes, either IgA, IgG or IgM (Anderson *et al.*, 1966). A survey carried out in Minnesota in residents aged 50 and over, showed a prevalence of 1.25% of benign IgM spikes, with a rate of 4.8% in the 80+ age group (Kyle *et al.*, 1972). Although the manifestation of allergies results from abnormal IgE production, with release of histamine from mast cells lining the respiratory and gastrointestinal tract (Wintrobe *et al.*, 1974), perhaps the immune system of patients with allergies are also predisposed to other plasma cell abnormalities.

The finding of an increased risk of myeloma among individuals employed in agriculture may indicate that some group of compounds associated with farming may act in conjunction with the natural tendency for an increased risk of developing BMG with age. It seems clear, however, that agricultural exposure may not be unique in this regard, as a number of studies have implicated asbestos (Robertson *et al.*, 1971), wood dust (Brinton *et al.*, 1976; Decouffle *et al.*, 1977), leather dust (Decouffle *et al.*, 1977) and heavy metal exposure (Decouffle *et al.*, 1977; Axelson *et al.*, 1978) as factors with possible associations with myeloma.

Further work is indicated on the relationship of allergies and myeloma. In particular, future studies should focus on separating the different types of hypersensitivity and better investigating the severity of allergies, and the allergens responsible for them. In addition, specific information on socio-economic status of the subjects is required to eliminate possible confounding by this variable.

Future aetiological studies should also specifically address the possible association between myeloma and farming by collecting detailed data on the type of agriculture in which the respondents are engaged.

The authors would like to acknowledge the aid of Ms. Joanne Moody and Ms. Margaret Fung with the study, and the secretarial assistance of Ms. Lynda Jeffries and Ms. Shirley Morton. This study was supported in part by Health & Welfare Canada NHRDP Grant No. 6610-1134-44.

### References

AGU, V.U., CHRISTENSEN, B.L. & BUFFLER, P.A. (1980). Geographic patterns of multiple myeloma: racial and industrial correlates, State of Texas, 1969–71. *J. Natl Cancer Inst.*, **65**, 735.

ALLEN, T.M. (1970). ABO blood groups and myelomatosis. *Br. Med. J.*, **4**, 178.

ANDERSON, U., BACHMANN, R. & HALLEN, J. (1966). Frequency of pathological proteins (M-components) in 6995 sera from an adult population. *Acta Med. Scand.*, **179**, 235.

AXELSON, O., DAHLGREN, E., JANSSON, C.D. & REHNLUND, S.O. (1978). Arsenic exposure and mortality: a case referent study from a Swedish copper smelter. *Br. J. Ind. Med.*, **35**, 8.

B.C. MINISTRY OF HEALTH. (1976). Cancer in British Columbia, 1969–73 Ministry of Health, B.C.

BLATTNER, W.A. (1980). Epidemiology of multiple myeloma and related plasma cell disorders: an analytic review, In *Progress in Myeloma* (ed. Potter) Elsevier North-Holland, New York. p. 65.

BRESLOW, N.E. & DAY, N.E. (1980). *Statistical Methods in Cancer Research Vol I* IARC Scientific Publication No. **32**, Lyons.

BRINTON, L.A., STONE, B.J., BLOT, W.J. & FRAUMENI, J.F. (1976). Nasal Cancer in U.S. furniture industry counties. *Lancet.*, **ii**, 628.

BURMEISTER, L.F. (1981). Cancer Mortality among Iowa Farmers, 1971–1978. *J. Natl Cancer Inst.*, **66**, 461.

CUZICK, J. (1981). Radiation induced myelomatosis, *N. Engl. J. Med.*, **304**, 204.

DECOUFFLE, P., STANISLAWEZYK, HOUTEN, L., BROSS, I.D.J. & VIADANA, E. (1977). *A Retrospective Survey of Cancer in Relation to Occupation.* DHEW (NIOSH) Publ. No. 77–178 Washington D.C.

DOLPHIN, G.W. (1976). A comparison of the observed and the expected cancers of the haematopoietic and lymphatic systems among workers at Windscale: a first report. *Natl Radiol. Prot. Board,* (NRPB-R54) Harwell.

KYLE, R.A., HENDERSON, E.S., RANDOLPH, V.L. & BUDGE, W.R. (1976). Multiple myeloma, acute leukaemia and Hodgkin's Disease occurrence in three of four family members. *Cancer,* **37**, 1496.

KYLE, R.A., FRANKELSTEIN, S., ELVEBACK, L.R. & KURLAND, L.T. (1972). Incidence of monoclonal proteins in a Minnesota community with a cluster of multiple myeloma. *Blood,* **40**, 719.

KRUPP, M.A. & CHATTON, M.J. (1982). *Current Medical Diagnoses and Treatment* Large Medical Publications, Los Altos, Ca.

LEWIS, E.B. (1963). Leukaemia multiple myeloma and aplastic anaemia in American radiologists. *Science,* **142**, 1492.

MALDONADO, J.E. & KYLE, R.A. (1974). Familial myeloma. Report of eight families and a study of serum proteins in their relatives. *Am. J. Med.*, **57**, 875.

MILHAM, S. (1976). *Occupational Mortality in Washington State, 1950–1971.* DHEW Publication (NIOSH) No. 76–175, Washington, D.C.

MILHAM, S. (1971). Leukaemia and multiple myeloma in farmers. *Am. J. Epidemiol.*, **94**, 307.

PRIESTER, W.A. & MASON, T.J. (1974). Human cancer mortality in relation to poultry population, by county, in 10 Southeastern states. *J. Natl Cancer Inst.*, **53**, 45.

REGISTRAR GENERAL'S DECENNIAL SUPPLEMENT. (1978). *Occupational Mortality, England and Wales 1970–1972,* HMSO, London.

ROBERTSON, M.A., HARRINGTON, J.S. & BRADSHAW, E. (1971). The cancer pattern in African gold miners. *Br. J. Cancer,* **25**, 395.

SCHAFER, A.I. & MILLER, J.B. (1979). Association of IgA multiple myeloma with pre-existing disease. *Br. J. Haematol.*, **41**, 19.

VALEZ, R., BERAL, V. & CUZICK, J. (1982). Increasing trends of multiple myeloma mortality in England and Wales: 1950–79: Are the changes real? *J. Natl Cancer Inst.*, **69**, 387.

WATERHOUSE, J. & MUIR, C. eds. (1976). *Cancer Incidence in Five Continents,* I.A.R.C., Lyon 1976.

WINTROBE, W., THORN, G., ADAMS, R., BRAUNWALD, E., ISSELBACHER, K.J. & PETERSDORF, R.G. (1974). *Harrison's Principles of Internal Medicine* 7th Edition, McGraw Hill, New York.